# Mitochondrial DNA Integrity: Role in Health and Disease

**DOI:** 10.3390/cells8020100

**Published:** 2019-01-29

**Authors:** Priyanka Sharma, Harini Sampath

**Affiliations:** Department of Nutritional Sciences and New Jersey Institute for Food, Nutrition, and Health, Rutgers University, New Brunswick, NJ 08901, USA; priyanka.s1909@gmail.com

**Keywords:** mitochondrial DNA, base excision repair, metabolic syndrome, neurodegenerative diseases, aging

## Abstract

As the primary cellular location for respiration and energy production, mitochondria serve in a critical capacity to the cell. Yet, by virtue of this very function of respiration, mitochondria are subject to constant oxidative stress that can damage one of the unique features of this organelle, its distinct genome. Damage to mitochondrial DNA (mtDNA) and loss of mitochondrial genome integrity is increasingly understood to play a role in the development of both severe early-onset maladies and chronic age-related diseases. In this article, we review the processes by which mtDNA integrity is maintained, with an emphasis on the repair of oxidative DNA lesions, and the cellular consequences of diminished mitochondrial genome stability.

## 1. Introduction

Coming from the Greek words for thread (mitos) and granules (khondros), mitochondria serve the essential function of energy generation, earning them the oft-used moniker of being the “powerhouses” of the cell. Through the coordinated processes of oxidative phosphorylation, mitochondria indeed generate most of the cellular adenosine triphosphate (ATP) and also establish the mitochondrial membrane potential, which is critical to their function. They accomplish these activities through the synergistic actions of over a thousand proteins, the majority of which are encoded in the nuclear genome and imported into the mitochondria [1]. However, mitochondria are a unique organelle in that they house their own genome, distinct from the nuclear genome. While this genome encodes only thirteen peptides that are involved in oxidative phosphorylation, [2] they are a critical component of the cellular energy production machinery. Much of our understanding of mitochondrial organization and function has arisen from the study of mitochondrial diseases. Apart from the better-described severe maladies of mitochondrial origin, it is now becoming clear that chronic accumulation of lower levels of mtDNA damage and decreases in mtDNA copy number are not only associated with the aging process, but may also be causally linked to age-related diseases such as neurodegeneration and diabetes [3,4,5,6]. Given their reparable nature, oxidative lesions represent an important target in our quest to mitigate the age-related disease burden. In this review, we summarize recent progress in our understanding of the maintenance of mtDNA integrity via DNA repair pathways that target oxidative damage and the cellular consequences of a loss of mtDNA integrity.

## 2. Mitochondrial DNA: Structure

**Figure 1:**
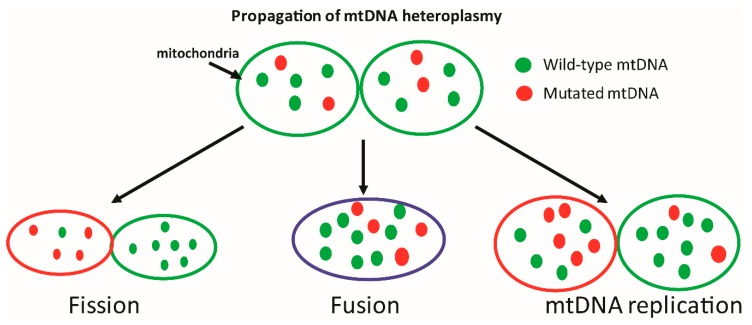
Mechanisms of inheritance of mtDNA mutations. Each mitochondrion consists of multiple copies of mtDNA, some of which may harbor harmful mutations. Upon mitochondrial fission, fusion, or mtDNA replication, these mtDNA molecules may be randomly segregated to daughter mitochondria, resulting in either reduced or increased levels of heteroplasmy. The contribution of heteroplasmy to disease development is difficult to study, as the disease threshold for each mutation may be different and may lead to a range of clinical and sub-clinical phenotypes. (Green ovals = functional mitochondria; red = dysfunctional; blue = suboptimal function).

mtDNA encodes for many of the essential components of the oxidative phosphorylation chain, and is thus crucial to ATP generation. Save for a couple of notable anomalies such as budding yeast that can survive anaerobic growing conditions and mtDNA-depleted cells that can subsist on glucose and glycolysis while in culture [7,8,9], mtDNA is essential to life. Interestingly, smaller non-life threatening mutations have also been shown to induce pathologies that may manifest later in the life-cycle or under conditions of metabolic stress [4,10,11,12]. The contribution of such mutations to cellular function is notably harder to decipher, since disease symptoms may exist on a spectrum which is dependent on the number of mitochondria harboring a given mutation. The existence of both healthy and mutated mtDNA within a given mitochondrion is referred to as heteroplasmy (Figure 1), and likely has a profound influence on cellular function [13]. Cellular tolerance of mtDNA heteroplasmy is a topic that is not completely understood, as tolerance is likely to (a) differ based upon the mutation and cell type, (b) be significantly influenced by competing processes such as mitochondrial biogenesis and degradation, and (c) result in a spectrum of phenotypes, many of which may be below clinical detection levels. 

Human mitochondrial DNA (mtDNA) is a double-stranded circular molecule of 16,569 base pairs with a molecular mass of 10^7^ daltons [2,14]. It is associated with proteins and organized in nucleoids located within the mitochondrial matrix, in close proximity to the mitochondrial membrane [2,15,16,17,18]. mtDNA encodes 13 polypeptides of the oxidative phosphorylation complex (OXPHOS) that are localized to the mitochondrial inner membrane. These include seven subunits of complex I, one subunit of complex III, three subunits of complex IV, and two of complex V, all of which are required for the generation of ATP by oxidative phosphorylation [2,11,15]. The vast majority of mitochondrial proteins, however, are encoded by the nuclear genome and are imported into the mitochondria by translocation systems localized to the outer and inner mitochondrial membranes [19]. The mitochondrial genome also encodes for 22 transfer RNAs and 2 ribosomal RNAs (12S and 16S rRNAs) for mitochondrial translation [2,20]. Unlike genomic DNA, mtDNA lacks histones and is maternally inherited [11,14,21,22], although evidence for partial paternal inheritance has recently been described [23]. 

The double-stranded structure of mtDNA consists of a guanine-rich heavy (H) strand and a light (L) strand [24]. The organization of mtDNA across vertebrates is similar to that of humans, with the majority of noncoding DNA being located within a ~1 kb region dubbed the noncoding region (NCR). The NCR is the most polymorphic site within mtDNA with several known polymorphisms within two hypervariable regions (HVR) in the NCR [25,26]. Sequencing of the HVRs of mtDNA can be used to ascribe mitochondrial haplogroups and is useful in tracing genetic lineages of human populations [27]. The noncoding region (NCR) serves a regulatory function within mtDNA, exerting control over transcription and translation. The mtDNA control region contains the replication origin for one strand and the transcription origin for both strands [20,28]. The control region is also the site of the displacement loop (D-loop) of mtDNA. While often used interchangeably, the D-loop only forms part of the control region and is not analogous to the NCR. The D-loop consists of a triple strand of DNA containing the heavy and light strands and a partially replicated heavy strand, hydrogen bonded to the light strand [29,30]. Replication of mtDNA begins in the D-loop, which also contains promoters of transcripts adjacent to the D-loop. While the exact function of the D-loop is unclear, it is noteworthy that this region is subject to high sequence variability [31] and has been shown to be associated with the incidence of particular types of cancers [32,33,34,35,36,37]. Mutations in the mitochondrial control region have also been associated with aging in skeletal muscle [38] and skin fibroblasts [39].

## 3. Mitochondrial DNA: Transcription and Replication

Apart from the NCR, the remainder of the mtDNA molecule consists of genes without intervening intronic structures and often with overlapping reading frames between adjacent genes [40]. Further, every mitochondrion can contain multiple copies of mtDNA, with cells carrying numerous functional mitochondria per cell. The compact organization of mtDNA, along with the presence of multiple copies of mtDNA within cells, may help explain the relatively high gene density of mtDNA. Despite the minuscule size of the mtDNA molecule relative to the nuclear genome, mRNA of mitochondrial origin can represent a large proportion of total cellular mRNA, ranging from 5% in the lung to up to 30% in the human heart [40,41]. 

mtDNA transcription is initiated from two promoters on the H-strand (HSP1 and HSP2) and one on the L-strand (LSP) [42,43,44]. Mitochondrial genes are transcribed as polycistrionic precursors that are subsequently processed into individual RNAs [28,42,45]. mtDNA is transcribed into three primary transcripts, the shortest of which originates at HSP1. The second transcript originates at HSP2 and continues for almost the entire length of mtDNA. The third transcript originating at the LSP is close to genomic length [42,46,47,48,49]. These primary transcripts are further processed via sequential processes of cleavage, polyadenylation, and tRNA and rRNA modifications [42]. While mtDNA abundance is clearly correlated with the abundance of mitochondrial transcripts, much of the regulatory effects on mitochondrial transcription are exerted by nuclear-encoded proteins, many of which are still being identified. mtDNA transcription was previously thought to be regulated solely by the mitochondrial RNA polymerase (POLRMT), two transcription factors (TFAM and TF2BM), an elongation factor (TEFM), and a transcription termination factor (MTERF1) [50,51,52]. However, more recent studies have indicated a role for other proteins that have previously been shown to regulate nuclear gene transcription [42,53,54,55,56,57]. Further, mitochondrial transcript abundance is also regulated by cellular ATP levels and rates of transcript turnover [42,58]. There is also some evidence to suggest that similar to genomic DNA, mtDNA methylation may play a role in regulating transcript abundance [42,59,60,61]. These, and other aspects of mitochondrial transcription, including the existence of two origins on the heavy strand, the relative strengths of each of the origins, and mechanisms mediating the relative abundance of transcripts originating from each of the sites are all areas of active investigation, as discussed in greater detail in a recent review [42]. mtDNA replication (Box 1) is a complex process, our understanding of which is ongoing. For a more comprehensive review of this evolving and fascinating topic, we direct the reader to several excellent reviews dedicated to mtDNA replication [25,40]. Briefly, mitochondria house a distinct DNA replication system that includes nuclear-encoded proteins such as DNA polymerase γ, TWINKLE helicase, mitochondrial single-stranded DNA binding protein (mtSSB), and POLRMT [62,63,64,65,66,67], as well as RNase H1, DNA ligase III, and topoisomerase 3α [68,69,70,71,72]. Despite using a similar cast of players, mtDNA replication is distinct from nuclear replication in many ways, including the fact that mtDNA replication occurs not only in dividing cells, but also in post-mitotic tissues and across all stages of the cell cycle. The most widely-accepted model for mtDNA replication involves a strand-displacement mechanism, wherein leading and lagging strand synthesis are uncoupled (Box 1) [73,74,75]. However, in recent years, competing models of mt DNA replication, including the RITOLS model and conventional coupled strand synthesis have also been suggested [76,77,78,79]. The precise mechanisms involved in these alternative mode of replication have been recently reviewed [25]. Defects in mtDNA replication proteins are associated with severe multi-system diseases such as ataxia-neuropathy [80]. Given the importance of accurate replication of its genome to mitochondrial function, the study of mtDNA replication is an active and critical area of inquiry.

While the processes of mitochondrial transcription and replication are well-studied, the impact of oxidative DNA lesions on the rates and fidelity of these two processes is far from clear. This topic is discussed below, following a brief review of mtDNA damage and repair pathways.

Box 1 **Mitochondrial DNA Replication—Competing Theories.**

In the more classical and widely-accepted model of mtDNA replication, the strand displacement model, single-stranded replication of the heavy (H) strand begins with further expansion of a nascent displacement loop(D-loop). This continues until the light (L) strand origin (O_L_) is exposed, after about two-thirds of the heavy strand has been replicated, when synthesis of the L-strand begins in the opposite direction. This asymmetrical strand synthesis results in one of two daughter molecules containing a partially-synthesized L-strand, until the replication process is completed. In an alternatively proposed strand-coupled or synchronous model of mtDNA replication, the replication process is initiated within the D-loop, with both strands being synthesized bidirectionally and simultaneously. In the RITOLS model, replication begins at one of two sites of origin. This is followed by leading strand synthesis with simultaneous incorporation of RNA on the lagging strand. Lagging strand synthesis is presumed to begin at O_L_, following which the lagging strand RNA is either converted to or replaced by DNA.

## 4. Induction of Mitochondrial DNA Damage

Given the indispensable role of mitochondrially-encoded proteins in regulating energy production in the cell, enzymes responsible for maintaining mtDNA stability and integrity are of critical importance to cellular energetics. The need to maintain mtDNA integrity is complicated by the fact that mtDNA is particularly vulnerable to damage, especially through the generation of oxidative lesions [81,82]. There are several potential reasons for this increased sensitivity of mtDNA to oxidative lesions. First, mtDNA resides in close proximity to the site of reactive oxygen species (ROS) production in the mitochondrial membrane. Second, mtDNA replication proceeds via an asymmetric route (Box 1), resulting in parts of the heavy strand existing as single-stranded structures for extended periods of time; this can lead to the spontaneous deamination of nucleotides [83,84]. Further, factors such as the asymmetric distribution of dNTPs in mitochondria, favoring higher levels of dGTP, can result in decreased fidelity during mtDNA replication, contributing to the higher rate of spontaneous mutagenesis in the mitochondrial genome [85]. By various estimates, mtDNA undergoes a high rate of mutagenesis, potentially 10- to 20-fold higher than that of the nuclear genome [86,87,88,89].

Both the process of respiration, as well as exogenous exposures, can result in damage to DNA bases [90]. Further, mutations in proteins involved in oxidative phosphorylation can also serve to increase cellular ROS level [91,92]. Mitochondrial respiration generates multiple reactive species, including superoxide, hydrogen peroxide, nitric oxide, peroxynitrite, hypochlorous acid, singlet oxygen, and the hydroxyl radical, which react with and damage cellular proteins, lipids, and DNA [81,82,93,94,95]. Cellular ROS levels are regulated by enzymatic antioxidants such as superoxide dismutase (SOD), glutathione peroxidase, and catalase [96,97,98], many of which are activated by the nuclear erythroid-2 like factor-2 transcription factor binding to antioxidant response elements in their promoter regions [99]. Mitochondrial ROS reduction is also achieved via proton leak across the mitochondrial membrane, a process regulated by uncoupling proteins at the expense of ATP generation [100]. Despite the existence of these numerous mechanisms, as well as cellular antioxidants such as ascorbate and tocopherols, ROS quenching is by no means a perfect process. ROS-induced damage results in base substitutions, missense mutations, and deletions within the mitochondrial genome, all of which impact mitochondrial function. mtDNA has been shown to be more prone to damage as a consequence of aging [89,101,102,103,104,105,106,107,108], potentially due to both increased ROS production as well as reduced ROS quenching, and decreased DNA repair capacity with increasing age [81,82,109,110]. mtDNA has also been shown to be damaged at lower ROS levels than genomic DNA. Further, repair of mtDNA is a slower process than genomic repair, especially following longer durations of oxidative stress [89,101,102,103,104,105,106,108]. 

Mitochondria co-opt nuclear DNA repair factors to repair mtDNA lesions via a number of DNA repair pathways, including mismatch repair and non-homologous end joining, which have been reviewed elsewhere [86,90,108,111]. However, the best characterized and predominant repair pathway in mitochondria is the base excision repair (BER) pathway. BER removes small non-helix distorting lesions such as oxidized and deaminated bases, alkylation damage, and single-strand breaks [112,113,114]. The importance of this pathway for the maintenance of mtDNA integrity is evidenced by the numerous pathological phenotypes described in human cohorts and animal models with defects in BER [81,82,115,116,117,118,119]. 

## 5. Base-Excision Repair of Mitochondrial DNA

**Figure 2:**
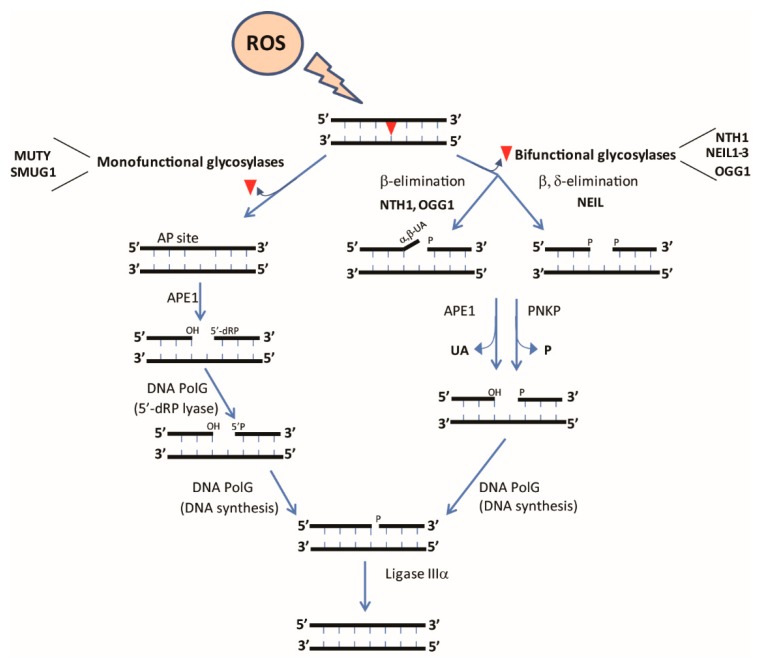
Base Excision Repair (BER) in mitochondria. Generation of free radicals induces DNA lesions in the form of oxidized bases, affecting base-pairing properties. Oxidative lesions are repaired via the BER pathway. BER is initiated by the activity of DNA glycosylases such as monofunctional glycosylases which recognize and cleave the *N*-glycosidic bond between the modified base and sugar, creating an abasic site and bifunctional glycosylases which have an additional intrinsic Apurinic/Apyrimidinic (AP)lyase activity. The incision of the AP site occurs via β elimination or β-δ elimination which is further processed by APE1 or PNKP, followed by gap-filling by DNA pol gamma (PolG). Once the AP site has been processed and the correct nucleotide recruited by PolG, the free DNA ends are ligated by DNA ligase III (LIG3).

BER is a multi-step pathway that can act via either short-patch (insertion of 1 nucleotide) or long-patch (insertion of 2–10 nucleotides) mechanisms. The overall steps of this pathway include (i) recognition and excision of the damaged base, (ii) removal of the abasic site, (iii) end processing, (iv) gap filling, and (v) DNA ligation (Figure 2). The structure and biochemistry of each of the enzymes involved in this multi-step pathway has been recently reviewed [108,120,121,122]. BER is initiated by DNA glycosylases that recognize specific DNA lesions and cleave the N-glycosidic bond between the damaged base and the DNA backbone [81,82,121]. DNA glycosylases can be divided into mono- or bifunctional glycosylases. Monofunctional glycosylases such as adenine DNA glycosylase (MUTY) possess only glycosylase activity, necessitating the activity of a downstream enzyme, AP endonuclease (APE1), to generate a single-strand break. Non-oxidative lesions such as deaminated and alkylated bases are primarily excised by monofunctional DNA glycosylases, with MUTY being responsible for excision of mispaired adenines across from oxidized guanines [123]. In contrast, most oxidative lesions are recognized by bifunctional glycosylases such as endonuclease III like-1 (NTH1) that possess both glycosylase and AP lyase activities. Base removal by bifunctional glycosylases requires further processing of DNA ends by APE1 and polynucleotide kinase phosphate (PNKP), followed by nucleotide insertion by POLG (or polymerase beta in the nucleus) [108,121,124,125,126]. 

DNA glycosylases are encoded in the nucleus with several enzymes or variants containing a mitochondrial targeting sequence (MTS) that allows for mitochondrial localization of the glycosylase (Table 1). Six DNA glycosylases have been described to repair oxidized DNA lesions in humans [81,82,108,121]. These include MUTY homolog (also called MYH), 8-oxoguanine DNA glycosylase (OGG1), endonuclease three homolog 1 (NTH1), and Nei endonuclease VIII-like 1, 2, and 3 (NEIL1, NEIL2, and NEIL3). Of these glycosylases, MUTY, OGG1, NEIL1, NEIL2, and NTH1 have thus far been detected in mitochondria (see Table 1 for references). 

As noted above, the enzyme MUTY is responsible for the cleavage of mispaired adenines opposite oxidized guanine (8-oxoG). MUTY is ubiquitously expressed in the human, with the highest expression levels reported in thymus and testis, as well as in embryonic tissues [127]. The NEIL family of glycosylases acts upon oxidized pyrimidines such as FapyA and G, hydroxyuracil, further oxidation products of 8-oxoG such as the hydantoin lesions, and thymine glycol, among other lesions. NEIL1 [128,129,130] is expressed ubiquitously with the highest expression reported in the liver, thymus, and pancreas. NEIL2 [131] has been reported to have the highest expression levels in the testes and skeletal muscle, and NEIL3 [130] has been reported to be selectively expressed in areas of the brain containing stem cells. NTH1 and OGG1 are bifunctional DNA glycosylases that recognize and repair thymine glycol and oxidized guanine (8-oxoG, FapyG) lesions, respectively [81,82]. NTH1 is ubiquitously expressed in rodents, with high levels of expression being reported in the heart and brain [130,132]. Human OGG1 (hOgg1) has been reported to be expressed at the highest levels in the thymus, testis, intestine, brain, and germinal centers of B cells, as well as in differentiated keratinocytes in the upper granular layer of the epidermis [81,82,112,113,133,134,135]. In addition to these DNA glycosylases that act on oxidized lesions, the enzyme MutT homolog 1 (MTH1), while strictly speaking not involved in BER, is also partially mitochondrially-localized (Table 1) [136,137,138] and likely contributes to sanitation of mitochondrial dNTP pools to suppress rates of oxidative lesion incorporation into mtDNA [139,140].

## 6. Impact of Oxidative Damage on Mitochondrial Replication and Transcription

Despite the high rate of oxidative damage to mtDNA, the significance of ROS-induced mitochondrial lesions to transcription and replication are unclear. POLG is known to be unable to replicate past single strand breaks. However, it can bypass 8-oxoG in an error-prone manner, following some degree of replication stalling [160,161,162]. This stalling at 8-oxoG sites was found to be increased under low cellular dNTP conditions, as might be expected in post-mitotic tissues, where mtDNA replication is still an active process [160]. Replication stalling at 8-oxoG was shown to be mediated by the proofreading function of POLG, pointing to the presumed mutagenicity of 8-oxoG [160]. With regard to mitochondrial transcription, POLRMT has been shown to be able to bypass oxidative lesions such as 8-oxoG and thymine glycol, albeit while generating transcripts containing G:C to T:A transversions [163,164,165,166]. This bypass was shown to be more efficient in the presence of a nuclear elongation factor, TFIIS [166,167]. More recently, it has been demonstrated that lesion bypass by POLRMT is enhanced in the presence of the TEFM, effectively reducing early transcript termination at 8-oxoG sites [163,164]. Thus, while the transcription of mtDNA containing oxidative lesions is likely to occur in vivo, the impact of such lesion bypasses on transcript sequences or the generation of aberrant proteins has, to our knowledge, not been systematically investigated. In particular, while several mitochondrial transcription and replication studies have been performed using reconstituted enzymes and in vitro systems [163,164,165,166,167], there is a marked paucity of data regarding the impact of mtDNA lesions on transcription in vivo. This is an especially important deficit, since differences in in vitro experimental systems, such as those using promoter-independent transcription vs. a more complete transcription system, have yielded divergent results with regard to error bypass [163]. This is a notable shortcoming of in vitro transcription systems, especially given that our understanding of the proteins involved in mitochondrial transcription is still evolving. 

In this regard, we recently demonstrated that mice with enhanced expression of mitochondrial OGG1 had an increase in mitochondrial transcript abundance under conditions of oxidative stress [116]. This resulted in an increase in mitochondrial OXPHOS subunits and enhanced oxidative respiration in these mice [116]. Although indirectly, these data argue for a role for OGG1 in potentially regulating the rate and fidelity of mitochondrial transcription. However, it is unclear if this increase in mitochondrial transcript formation is due to a reduction in 8-oxoG content, an overall reduction in oxidative stress, or due to an as yet unidentified role of OGG1 in regulating mitochondrial transcription. Given the importance of mitochondrial replication and transcription to cellular metabolism, further studies in this area, particularly using in vivo models, are warranted.

## 7. Pathologies Associated with Mitochondrial Repair Defects

While the structure, mode of action, and biochemical regulation of these DNA glycosylases has been extensively studied, we know far less about the physiological relevance of unrepaired mtDNA damage to the development of disease. Given the known correlation between aging and the accumulation of DNA damage, perhaps it is not surprising that many of the pathologies associated with defects in BER can be considered diseases of aging. Through the study of human polymorphic variants and rodent models with engineered defects in glycosylase function, it has become evident that the efficient repair of oxidative lesions in genomic and mtDNA is integral to the prevention of cancers, neurodegenerative diseases, and metabolic pathologies. The role of genomic DNA damage in the progression of chronic and age-related diseases has been reviewed elsewhere by us and others [81,82,168,169,170,171]. The section below will focus on our current, more limited understanding of the role of mtDNA damage and repair in disease progression.

mtDNA mutations can be categorized into four broad classes, namely polypeptide mutations, rRNA and tRNA mutations that result in defects in protein synthesis, rearrangement mutations, and mutations within the control region that can impact mtDNA replication and transcription. Using databases of mtDNA sequence and variation such as the comprehensive MITOMAP initiative [172], a number of associations between specific mitochondrial mutations and proclivity or protection from disease have been reported. For instance, mtDNA mutations and deletions are implicated in many severe diseases (Table 2), including Mitochondrial Encephalopathy, Lactic acidosis, and Stroke-like episodes (MELAS), Kearns-Sayre Syndrome (KSS), Pearson syndrome, and in about 20% of cases of Leigh syndrome [173,174,175,176]. Like many severe maladies of mitochondrial origin, MELAS is typically an early-onset disease, with most patients developing symptoms before age twenty. MELAS is characterized by seizures, headaches, and stroke [173]. KSS is another early-onset neuromuscular disease characterized by pigmentary retinopathy and cardiac conduction defects, among other symptoms [176]. Pearson syndrome is often fatal in infancy and is characterized by exocrine pancreas insufficiency and sideroblastic anemia [174]. Leigh syndrome, a severe neurological disorder, is often diagnosed within the first year of life and generally results in respiratory failure and death within two to three years [175]. Other diseases of known mitochondrial origin include Leber hereditary optic atrophy [177] and a type of epilepsy called MERFF (Myoclonus Epilepsy with Ragged Red Fibers), among others [178].

Apart from these severe maladies, it is becoming increasingly clear that several chronic age-related diseases are also related to impaired mitochondrial function [183,184]. While mitochondrial deficits do not necessarily originate within the mitochondria, mutations within mtDNA and alterations in mtDNA repair enzymes can be a direct cause of age-related pathologies [183,184]. 

## 8. Cancer

Defects in mitochondrial function have long been proposed to be involved in the etiology of cancers. Cancerous cells undergo profound alterations in oxidative metabolism, resulting in reduced flux through mitochondrial energy generating pathways [185]. Since mtDNA encodes for several critical proteins of the respiratory chain, it is highly plausible that mutations in mtDNA may impact the respiratory function of cells and thereby contribute, if not to cancer initiation, at least to propagation of the metabolic phenotype of cancer cells. However, unraveling the role of mtDNA mutations and deletions in regulating cellular metabolism is complicated by several factors, including the presence of high levels of heteroplasmy, the use of inappropriate reference sequences for control groups in previously published studies, and the complexity of segregating primary ‘driver’ mutations from secondary ‘passenger’ mutations [186,187]. Further, since many described mitochondrial mutations associated with cancers do not induce amino acid changes, the role of such mutations to carcinogenesis is not clear [10,188]. 

Despite these caveats, several specific mutations and deletions of mtDNA affecting both the control and coding regions have been reported in various types of cancers [10]. For instance, a 264 bp deletion in the region encoding for NADH:ubiquinone oxidoreductase (complex I) of the electron transport chain has been demonstrated to be associated with renal cell carcinoma [189]. In another notable example, a T8993G mutation in the coding region of the *Atp6* mitochondrial gene has been reported in a prostate cancer cell line. This results not only in reduced levels of ATP6 protein, but also in increased ROS production that could presumably serve to perpetuate the oxidative DNA damage cycle [188]. In this particular example, the mutation results in an amino acid change in an integral respiratory chain protein and is associated with increased tumor size. However, to our knowledge, no one has demonstrated why these and other mitochondrial mutations are increased in tumors or cancer cells, and also, whether they are drivers of the cancer phenotype.

## 9. Neurodegenerative Diseases: Parkinson’s and Alzheimer’s

Oxidative stress and accumulation of oxidative damage is associated with neurodegenerative diseases [82,168,169,190,191,192]. However, a clear understanding of mtDNA damage and repair being mechanistically linked to the onset or progression of neurodegeneration, including Parkinson’s and Alzheimer’s disease, is only beginning to emerge. In this regard, neural stem cells (NSCs) from OGG1-deficient (*Ogg1^−/−^*) mice have been shown to accumulate more mtDNA damage, committing them to an astrocytic lineage at the expense of neurogenesis [193]. Treatment of these NSCs with antioxidants, or the introduction of a mitochondrially-targeted OGG1 protein ameliorated mtDNA damage and rescued neurogenesis, indicating the importance of mtDNA repair to NSC fate determination [193].

Studies using mice deficient for OGG1 and MTH1 have demonstrated that double-knockout mice suffer from severe striatal neurodegeneration [194]. Cortical neurons isolated from double-knockout mice demonstrated poor neurite outgrowth, secondary to increased mtDNA damage, especially in the absence of exogenous antioxidants in the culture media [195]. Interestingly, the increase in neuronal stress in the absence of OGG1 and MTH1 is likely a consequence of MUTY-mediated repair of mispaired adenines across 8-oxoG sites [194]. Deletion of MUTY effectively abrogated the neurodegeneration observed in *Ogg1^−/−^* mice [194]. Similarly, transgenic overexpression of human MTH1 also reduced mtDNA 8-oxoG accumulation and efficiently prevented neurodegeneration in OGG1-deficient animals. Apart from these studies using rodent models of DNA repair defects, studies of human cohorts have suggested that carriers of the mitochondrial haplogroup H5 may have an increased risk for Alzheimer’s disease [196].

Higher levels of mtDNA deletions have been reported in the striatum of Parkinson’s patients, relative to unaffected age-matched controls [27,197,198]. Mutations reported to be associated with Parkinsonism include those in mitochondrially-encoded subunits of complex I [197]. Interestingly, defects in PolG have also been reported to be associated with Parkinsonism in some families [199]. There have been mixed reports about a role for mitochondrial repair enzymes such as OGG1, APE1, and NEIL1 in modulating risk for Parkinson’s, either as a stand-alone risk factor or in combination with other exposures [137,200,201,202,203,204]. However, more studies are required to determine if specific defects in the mitochondrial forms of these enzymes represent a causative risk for development of Parkinson’s.

## 10. Metabolic Disease

Chronic consumption of hypercaloric diets, including those high in fat or simple sugars, is causatively linked to the development of obesity and related metabolic derangements, including fatty liver disease, type-2 diabetes, adipose tissue inflammation, and cardiovascular disease. While increased caloric intake is a recognized source of oxidative stress, the role of DNA repair enzymes in regulating metabolic disease had not been explored until recently. In this regard, the first demonstration of a BER enzyme being important to the prevention of metabolic disorders came from mice lacking the BER glycosylase, NEIL1 (*Neil1^−/−^*). *Neil1^−/−^* mice are prone to obesity and glucose intolerance [118,141]. Chronic high-fat diet further impaired metabolic function in these mice, secondary to an increase in mtDNA deletions and reduced mitochondrial protein content [118,141]. Similarly, engineered deletion of NEIL3 has been recently shown to increase susceptibility to atherosclerosis and increased size of atherosclerotic plaques in hypercholesterolemic mice deficient for the apolipoprotein, APOE3 [205]. The role of mitochondrial NEIL1 vs. the nuclear form of the enzyme in mediating metabolic phenotypes has not yet been determined, and NEIL3 does not have any reported mitochondrial localization.

In a similar, better-characterized paradigm, mice deficient for the BER glycosylase OGG1 are prone to obesity and insulin resistance, due to a specific reduction in fatty acid oxidation [117]. More recently, OGG1-deficient mice were shown to have impaired control over hepatic gluconeogenesis [206]. Our group also reported that OGG1-deficient mice have increased ectopic lipid accumulation in skeletal muscle, associated with increased mitochondrial fission and accelerated muscle function decline [115]. OGG1, like other DNA glycosylases, has been shown to have both nuclear and mitochondrial localization [82]. While the -2a or β isoform of OGG1 was initially presumed to be the main mitochondrial isoform [135], a recent study demonstrated that the endogenous mitochondrial-targeting sequence of the predominant -1a isoform is sufficient to target both the nucleus and mitochondria [151]. In prior experiments using cells in culture, several reports established a protective role for mitochondrial OGG1 in lipid-induced insulin resistance and apoptosis [146,147,148,207]. A recent study from our lab demonstrated that mitochondrial-targeting of OGG1 confers significant protection against diet-induced obesity and insulin resistance, even in the complete absence of nuclear OGG1 [116]. This metabolic protection occurs secondary to increased mitochondrial protein content and increased mitochondrial length, that combine to enhance mitochondrial respiration and whole-body energy expenditure [116]. These data are the first demonstration of mitochondrial repair of oxidative DNA lesions being sufficient to protect animals against the adverse outcomes of chronic high-fat diet consumption. It is notable that in this model of OGG1 mitochondrial overexpression, multiple metabolic parameters were affected primarily in white adipose tissue [116]. This was accompanied by a reduction in 8-oxoG levels in adipose tissue of these transgenic mice, indicating that the DNA repair activity of OGG1 likely mediates the metabolic protection observed in these mice. A previous study that reported on overexpression of the -2a isoform of OGG1 reported no protection against metabolic disease in this model [208]. This isoform of OGG1 has been reported to be devoid of DNA glycosylase activity [208], thereby lending further support to the hypothesis that the DNA-repair functionality of mitochondrial OGG1 is required for its metabolic effects [116]. 

In human populations, a relatively common OGG1 polymorphism, Ser326Cys, is associated with reduced glycosylase activity, and has been shown to be correlated with increased body mass index and elevated total cholesterol and fasting blood glucose in Japanese individuals homozygous for the mutation [209]. Similarly, in a Mexican cohort, the Ser326Cys polymorphism was independently correlated with increased incidence of type-2 diabetes [119]. Further, in a recent report from the European PREDIMED study, individuals homozygous for the Cys/Cys genotype had increased total mortality, attributable to cardiovascular disease-related deaths, but not to cancer-related mortality [210]. Together, these data in human populations underscore a role for OGG1 in modulating metabolic risk. Given our recent report of mitochondrial OGG1 being protective against obesity and metabolic disease [116], it will be interesting to determine if alterations in mtDNA integrity and mitochondrial function may be observed in carriers of the Ser326Cys mutation.

## 11. Summary

While mitochondrial function has long been understood to be critical to the health of the cell, the complex topic of mitochondrial origins of disease is only beginning to be explored. In this regard, the study of mtDNA integrity is challenging due to, among other factors, the lower abundance of mtDNA relative to genomic DNA and the existence of varying levels of heteroplasmy that may contribute to disease. However, despite these challenges, our understanding of mitochondrial genome integrity as a driver of health and disease continues to expand. Further, other mitochondrial processes including the regulation of mitochondrial biogenesis, maintenance of mitochondrial networks via fission and fusion pathways, the process of selective degradation of defective mitochondria via mitophagy, and interorganelle communication between mitochondria and organelles such as the endoplasmic reticulum are all increasingly understood to be vital to cellular respiration and energy production. Whether or not these processes are also regulated by underlying changes in mtDNA integrity is likely to be a fascinating novel area of investigation in upcoming years. 

## Figures and Tables

**Table 1:** Tissue specificity, cellular localization, and known or inferred functions of mitochondrial isoforms of selected BER glycosylases and MTH1.

Enzyme	Glycosylase Family	Tissue Specificity	Inferred or Confirmed Function(s) of Mitochondrial Isoform	Mitochondrial Localization Described
NEIL1	Fpg/Nei Helix-two turns-helix	Liver, thymus, pancreas, brain [128,129,130]	Potential role in mediating metabolic syndrome in *Neil1^−/−^* mice [141]; Binding partner for mtSSB [142]	[142,143]
NEIL2	Fpg/Nei Helix-two turns-helix	Testes and skeletal muscle [131]	Removal of oxidized bases from mitochondrial genome [144]	[144]
OGG1 (1a)	Helix-hairpin- helix	Thymus, testis, intestine, brain, and germinal center of B cells [130,133,135,145]	Role in protecting against oxidative stress [146,147,148,149]; Prevention of metabolic syndrome [116]	[150,151,152]
NTH1	Helix-hairpin- helix	Heart, brain [130,132,153]	Unknown; potentially compensated for by NEIL1 activity	Mouse isoform is exclusively mitochondrial; human isoform is thought to be exclusively nuclear [154,155]
MUTY	Helix-hairpin- helix	Thymus, intestine, heart, lung [156]	Potentially involved in repair of hypoxia induced damage in brain [157]	[156,158]
MTH1	Oxidized purine nucleoside triphosphatase	Thymus, testis, embryonic tissues [127]	Protection from oxidative damage in models of Parkinson’s disease [137]	[136,138,159]

**Table 2:** Mitochondrial diseases and their genomic localization.

Disease	Clinical Manifestations	Location
Leber Hereditary Optic Neuropathy (LHON)	Bilateral, painless subacute visual failure	Mutations in MT-ND1, MT-ND4 or MT-ND6 gene [179,180]
Myoclonic Epilepsy with Ragged Red Fibers (MERRF)	Myoclonus epilepsy, ataxia, muscle weakness and dementia	Mutations in MT-TK encoding tRNA lysine (tRNALys). A-to-G transition at nucleotide 8344 [178,181]
Pearson’s syndrome	Sideroblastic anemia and exocrine pancreas dysfunction	Mitochondrial DNA deletion [174,182]
Kearns-Sayre Syndrome (KSS)	Pigmentary retinopathy	Mitochondrial DNA deletion [176]
Mitochondrial encephalopathy, lactic acidosis and strokelike episodes (MELAS)	Mitochondrial encephalomyopathy, lactic acidosis and strokelike episodes. Other features include headache, seizures, muscle weakness	Point mutation in the tRNA leucine^UUR^ gene of mitochondrial DNA. A to G transition at nucleotide 3243 [173]
